# Changing Healthcare Policies: Implications for Income, Education, and Health Disparity

**DOI:** 10.3389/fpubh.2015.00195

**Published:** 2015-08-11

**Authors:** Tetsuji Yamada, Chia-Ching Chen, J. J. Naddeo, Joseph R. Harris

**Affiliations:** ^1^Department of Economics, Rutgers University Camden, Camden, NJ, USA; ^2^Center for Children and Childhood Studies, Rutgers University Camden, Camden, NJ, USA; ^3^Department of Epidemiology and Community Health, New York Medical College, Valhalla, NY, USA; ^4^Division of Federal-State Cooperative Programs, Bureau of Labor Statistics, U.S. Department of Labor, Philadelphia, PA, USA

**Keywords:** income effect, education effect, health disparity

## Introduction

The economic pie has seen growth, despite the recessions of the past decade, and has resulted in an increase in the total income level. At the same time, increasing national healthcare spending due to the high costs of medical research and development paired with a growing and aging population has become prevalent in most nations. Healthcare costs (HCs) are a significant factor that influences the current and future government budget allocations. These HCs are generating major problems for welfare systems in both the short and long term.

Income disparity has become a major issue in the past decade (1–3). Ettner (4) shows that increased income levels improve mental and physical health, and Wildman (5) theoretically proves the relationship between the income differential and health disparity (4, 5). Also, Liu et al. (6) document the health inequality implications of the increasing gap in income and healthcare utilization (6). Apart from income, education also has a positive effect on healthcare consumption and health status (7). Literature shows that increase in education enhances health capital. However, there is no clear discussion on the systematic relationship between the health educational differential and income level with health disparity (8).

This paper demonstrates how health education and income differentials affect health status thus creating health disparity.

## Healthcare

Under the government health insurance programs: Medicare, Medicaid, and State Child Health Insurance (SCHIP), which are designed to ameliorate unequal access to healthcare services caused by income inequality, governments tried to initiate programs that caped rising healthcare expenditures. The government partially implemented a policy change that switched from a cost-based reimbursement system, the fee-for-service system (FFS), to a capitation scheme (CS). In the US, there was a private and government oriented mixed financing system that caused the emergence of managed care plans, e.g., HMO, PPO, POS, etc. The policies addressed the efficiency of resource allocation and have decreased expenditures without affecting healthcare services utilization. The FFS payments include co-insurance with deductibles, exclusions and limits on covered benefits, and lifetime spending caps. The CS generally includes co-payments, sometimes with deductibles, and/or spending utilization caps.

The recent economic downturn has caused a sharp decrease in employer-provided health insurance benefits. The U.S. has a mixed healthcare system that has left a large portion of the population uninsured or underinsured. This leads to racial and socioeconomic driven healthcare access disparities, which are strongly associated with health outcome inequalities (2). As discussed earlier, the U.S. implements a mixed healthcare system where citizens can receive insurance privately or from the government. Medicaid is provided for low-income individuals by state governments, and Medicare is made available to retired citizens and is financed by the federal government. There are two parts to Medicare benefits: (1) a hospital insurance plan and (2) a physician insurance plan. State governments also fund the SCHIP, which was designed to reduce the number of children without adequate health insurance coverage for low-income families that do not qualify to receive Medicaid.

## Theoretical Framework: Diagrammatic Presentation

Assume that a society consists of two identical individuals. One individual’s preferences or consumption does not enter another individual’s utility function and preferences are not necessarily homothetic. Their demand functions are homogeneous of the degree 0 in income and prices. The relative prices of goods affect their consumption combination through changes in the production mix. The general increase in price level does not change the consumption bundle because of relative pricing. Both individuals produce health and a composite good, and both their income levels vary by health status. The efficient production set is non-linear, and their capital–labor ratios are different. Health as a good is relatively more capital intensive than composite goods. Also, there are no externalities in production.

**Figure 1:**
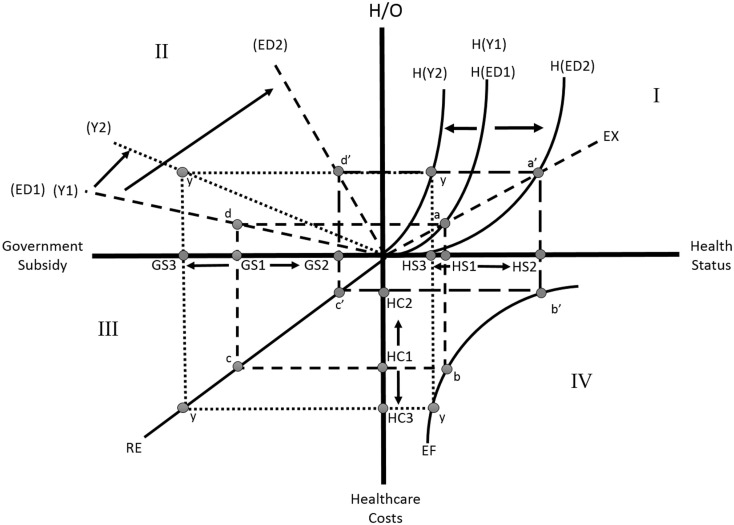
**Graphical representation of the effect of income and education on various health disparity indicators**.

Focusing on Quadrant I in Figure 1, the H(ED1) curve is derived from the marginal rates of transformation of the efficient production sets of Health (H) and composite goods (O), given the level of resources and technology (H/O). Both production functions H and O have constant returns but different capital–labor ratios along a non-linear efficient production set. A relative price of both goods rises along H(ED1) such that the unique price ratio of goods H and O equals the marginal rate of substitution. The H curve becomes vertical at the maximum level of relative consumption of H/O; it is steeper with a larger production of Health relative to the composite good due to the initial capital endowment.

We assume that as a function of H/O, the demand or choice of the combination of health and composite goods in the private sector is affected by a change in the relative prices. For healthcare production efficiency on the EF curve in Quadrant IV, there are two ways to evaluate efficiency: efficient resource allocation and efficiency of technological development to deliver healthcare services. The EF line shows that a decrease in health status, which moves health status from HS2 toward the origin (HS3) on Health Status horizontal line, increases HCs from HC2, HC1 to HC3. Efficiency of healthcare service production in Quadrant IV (EF line), which shows a negative and non-linear relationship between health status (HS) and HCs, does not affect the H/O level.

An increase in health education shifts the H line from H(ED1) to H(ED2) and raises health status from HS1 to HS2. The health educational effect goes from Quadrant VI through to Quadrant III. An increase in health status reduces HCs from HC1 to HC2 and reduces the government subsidy for an individual from GS1 to GS2 in Quadrant III through the RE. The RE in this quadrant indicates the reimbursement rate of the government to healthcare service providers. A rise in RE rotates this line toward the HC axes thus making it a steeper line.

Quadrant II shows that a more health-educated individual needs less government subsidy. The subsidy correspondingly declines from GS1 to GS2, because people with a higher level of education tend to lead healthier life styles. Better health-educated people utilize health and other market inputs, and their own time to produce a greater health output. An increase in health education, coupled with the improvement in health status and human capital, positively impacts the H/O level from d to d′ in Quadrant II. This increase would greatly benefit the society.

Another critical rationale on Figure 1, a decrease in income level would pivot the health status curve H from H(Y1) to H(Y2). Opposite results can be drawn for the EF and RE curves, in Quadrants IV and III, respectively. At the lower health level H(S3) due to a decrease in income H/O will be at y, implying that lower income will induce a lower level of health status. The reduction in income level will tend to decrease the availability/accessibility of healthcare services and in turn lower health stock. Thus, HCs inefficiently rise from HC1 to HC3 along the axis in Quadrant IV, and the government subsidy would rise from G(S1) to G(S3) with given y level of H/O. This situation is analogous to an increase in income tax.

## Results and Discussion

The data used in this study are sourced from the Behavioral Risk Factor Surveillance System (BRFSS) of 2013, a collaborative survey administered by every state in the United States. The BRFSS, an ongoing surveillance system designed to measure behavioral risk factors for the non-institutionalized population, is overseen and supported by the Center for Disease Control and Prevention (CDC). Some of the factors tracked by the BRFSS include substance abuse, HIV/AIDS prevention, physical activity, immunization, health status, preventive care, sleep, cholesterol and hypertension awareness and prevention, fruit and vegetable consumption, as well as various socioeconomic information. The original sample contained ~500,000 observations. After careful cleaning and application of age constraints (e.g., 18–64 years old), 24,300 observations remained.

The previous discussion in Theoretical Framework: Diagrammatic Presentation implies an increase in education or health education raises general health, namely health stock, in the long run. This shift in health stock will decrease the use of healthcare services, thus reducing healthcare costs. We used statistics method of ordinary least squares and paired its results with the elasticity concept in health economic theory. We found that a 1% increase in health education will lead to a certain percent increase in healthy days. Our estimation shows a 10% increase in education corresponds to a decrease in poor healthy physical and mental days by 24.3% (≈−2.64 × 4.85/5.27)10, which is {[coefficient of education × (mean of education/mean of poor healthy days)] × 10}. This shift from HS1 to HS2 is visually depicted in Quadrant I of Figure 1. We also evaluated the effect of education on physician visits. An increase in education by 10% lowers physician visits per year by 3.60% (≈−8.32 × 0.22/5.08)10, which is {[coefficient of physician visits × (mean of education/mean of physician visits)] × 10}. This decrease in physician visits decreases overall HCs, the shift from HC1 to HC2 is shown in Quadrant IV of Figure 1.

Both general and health education increases a person’s ability to read health information and understand preventative measures, thus increasing health stock (9). The results are consistent with our theoretical hypothesis, which is presented in Figure 1. In addition to education, income plays a role in a person’s general health. For example in Quadrant I, our study illustrates that a decrease in income by a 10% causes a decrease in mental and physical healthy days from HS1 to HS3 by 13.0% (≈−1.23 × 5.59/5.27)10, which is {[coefficient of healthy days × (mean of income/mean of healthy days)] × 10}. This income decrease also increases the number of physician visits per year by 12.3% (≈0.112 × 5.59/5.08)10, which is {[coefficient of income × (mean of income/mean of physician visits)] × 10}, and is shown in the movement from HC1 to HC3 in Quadrant IV. Estimates show that, in an optimal case, a healthy person visits their physician once or twice per year.

The concentration index [CI] is implemented in this study to measure health inequality (10). The index ranges between 0 and 1. A low index indicates more equality or equal distribution, while a high index indicates more disparity or unequal distribution. Our results show that the financial burden of healthcare falls disproportionately on the unhealthy segment of the population, i.e., health disparity (CI = 0.48). This can be explained by the fact that people with poor health also excessively visit the doctor, as shown in CI of the physician visit sector (CI = 0.26).

As stated previously, health education increases a person’s ability to obtain, process, and understand the basic health knowledge and information needed to make appropriate health decisions. Limited health knowledge is an enormous cost burden on government healthcare systems and increases the risk of errors in medication, patient compliance, and treatment.

Healthcare financing has a significant impact on health inequality. Healthcare costs/expenses are major obstacles for healthcare accessibility. It is imperative to develop a public healthcare financing system for the population that promotes equality. A recent increase in healthcare costs can be traced to an increase in access disparity. It is already known that worsening economic factors such as decreasing income are debilitating for the health of population.

The education variable in this study supports the hypothesis that formal and informal health education will lead to a more healthy population in the long run. In the short run, government led preventive care is a viable option that should be explored. It is essential for policy makers to make healthcare more affordable and accessible in order to reduce general healthcare inequality and lessen the overall healthcare-cost burden.

## Conflict of Interest Statement

The authors declare that the research was conducted in the absence of any commercial or financial relationships that could be construed as a potential conflict of interest.
